# Comparison on genomic predictions using three GBLUP methods and two single-step blending methods in the Nordic Holstein population

**DOI:** 10.1186/1297-9686-44-8

**Published:** 2012-07-06

**Authors:** Hongding Gao, Ole F Christensen, Per Madsen, Ulrik S Nielsen, Yuan Zhang, Mogens S Lund, Guosheng Su

**Affiliations:** 1 Department of Molecular Biology and Genetics, Aarhus University, DK-8830, Tjele, Denmark; 2 Danish Agricultural Advisory Service, DK-8200, Aarhus N, Denmark; 3 College of Animal Science and Technology, China Agricultural University, 100193, Beijing, People Republic of China

## Abstract

**Background:**

A single-step blending approach allows genomic prediction using information of genotyped and non-genotyped animals simultaneously. However, the combined relationship matrix in a single-step method may need to be adjusted because marker-based and pedigree-based relationship matrices may not be on the same scale. The same may apply when a GBLUP model includes both genomic breeding values and residual polygenic effects. The objective of this study was to compare single-step blending methods and GBLUP methods with and without adjustment of the genomic relationship matrix for genomic prediction of 16 traits in the Nordic Holstein population.

**Methods:**

The data consisted of de-regressed proofs (DRP) for 5 214 genotyped and 9 374 non-genotyped bulls. The bulls were divided into a training and a validation population by birth date, October 1, 2001. Five approaches for genomic prediction were used: 1) a simple GBLUP method, 2) a GBLUP method with a polygenic effect, 3) an adjusted GBLUP method with a polygenic effect, 4) a single-step blending method, and 5) an adjusted single-step blending method. In the adjusted GBLUP and single-step methods, the genomic relationship matrix was adjusted for the difference of scale between the genomic and the pedigree relationship matrices. A set of weights on the pedigree relationship matrix (ranging from 0.05 to 0.40) was used to build the combined relationship matrix in the single-step blending method and the GBLUP method with a polygenetic effect.

**Results:**

Averaged over the 16 traits, reliabilities of genomic breeding values predicted using the GBLUP method with a polygenic effect (relative weight of 0.20) were 0.3% higher than reliabilities from the simple GBLUP method (without a polygenic effect). The adjusted single-step blending and original single-step blending methods (relative weight of 0.20) had average reliabilities that were 2.1% and 1.8% higher than the simple GBLUP method, respectively. In addition, the GBLUP method with a polygenic effect led to less bias of genomic predictions than the simple GBLUP method, and both single-step blending methods yielded less bias of predictions than all GBLUP methods.

**Conclusions:**

The single-step blending method is an appealing approach for practical genomic prediction in dairy cattle. Genomic prediction from the single-step blending method can be improved by adjusting the scale of the genomic relationship matrix.

## Background

Selection based on dense markers across the genome [1] has become an important component of dairy cattle breeding programs [2-7]. The accuracy of genomic prediction relies on the amount of information used to derive the prediction equation. In many genomic selection programs, thousands of bulls which have been progeny tested over the last decades have been genotyped and are used as national reference populations. These have been extended by sharing data across countries to include much more information, such as the North American cooperation [8], the EuroGenomics project [7], and the joint Brown Swiss project [9]. Generally, genomic predictions are based on the data of all genotyped animals. However, in practice, not all individuals can be genotyped. To make use of as much information as possible for genetic evaluation, it is appealing to blend the genomic predicted breeding value and the traditional estimated breeding values (EBV) into genomically enhanced breeding values (GEBV) or to perform genomic prediction using all information available simultaneously.

Many studies have shown that a linear model which assumes that effects of all single nucleotide polymorphisms (SNP) are normally distributed with equal variance performs as well as variable selection models for most traits in dairy cattle [2,4]. Because such BLUP models are simple and have low computational requirements, they have become popular approaches for practical genomic prediction. De-regressed proofs (DRP) [10,11] are generally used as the response variable for genomic prediction since they can be easily derived from the EBV that are usually available.

Several blending strategies, including multi-step and single-step approaches, have been proposed to estimate GEBV [4,5,12-18]. The core of a single-step procedure is the integration of the marker-based relationship matrix into the pedigree-based relationship matrix such that information of genotyped and non-genotyped animals is used simultaneously [13-15]. Previous study by Su et al. [18] reported that a single-step procedure resulted in more accurate GEBV than a multi-step procedure.

Some studies [13-15,18] have reported that the combined relationship matrix in a single-step method may need to be adjusted because the marker- and pedigree-based relationship matrices may not be on the same scale, and different methods to adjust for this have been proposed [19-22]. These adjustments may also benefit genomic prediction using other models that integrate marker- and pedigree-based relationship matrices, such as a GBLUP model with a polygenic effect.

The purpose of this study was to compare single-step blending and GBLUP methods with and without adjustment of the genomic relationship matrix for genomic prediction of 16 traits in the Nordic Holstein population. De-regressed proofs were used as response variables in both GBLUP and the single-step blending methods.

## Methods

### Data

Data consisted of 5 214 genotyped bulls born between 1974 and 2008 and 9 374 non-genotyped bulls born between 1950 and 2008. The bulls were divided into a training and a validation population by birth date, October 1, 2001. Thus, the training data contained 3 045 genotyped and 8 822 non-genotyped bulls born before this date, and the validation data contained 2 169 genotyped bulls born after this date. Non-genotyped bulls born after October 1, 2001 were not used in training or validation. For the GBLUP methods described below, the training data only included the 3 045 genotyped animals. All 16 traits (sub-indices) in the Nordic Total Merit index were assessed, including yield, conformation, fertility, and health traits. For each trait, the DRP with reliability less than 0.20 were excluded from the training and the validation data. This removed 1.3%, 2.8% and 3.2% of DRP for birth index, fertility and health, respectively, and less than 0.5% for the other traits. The numbers of individuals in the training and validation datasets differed between traits (Table 1).

**Table 1:** **Heritability (h**^**2**^**) of the traits, number of bulls in training (Train) and validation datasets (Valid**_**gen**_**) for GBLUP and single-step blending**

**Trait**	**h**^**2**^	**Train**_**GBLUP**_	**Train**_**single**_	**Dif**^**1**^	**Valid**_**gen**_^**2**^
Milk	0.39	3003	9137	6134	1395
Fat	0.39	3003	9137	6134	1395
Protein	0.39	3003	9137	6134	1395
Growth	0.30	2538	6690	4152	1640
Fertility	0.04	3037	10909	7872	1378
Birth index	0.06	3045	10586	7541	2167
Calving index	0.03	3040	11538	8498	1501
Mastitis	0.04	3006	9174	6168	1461
Health	0.02	3026	9050	6024	1214
Body conf.	0.30	2884	7492	4608	1380
Feet & Leg	0.10	2925	7727	4802	1379
Udder conf.	0.25	2928	7743	4815	1380
Milkingspeed	0.26	2928	7725	4797	1380
Temperament	0.13	2926	7691	4765	1371
Longevity	0.10	2980	8740	5760	916
Yield	0.39	3003	9137	6134	1395

^1^Number of additional non-genotyped bulls used in single-step blending compared to GBLUP (Col.4 – Col.3); ^2^ Only genotyped bulls in the validation dataset.

Marker genotypes were obtained using the Illumina Bovine SNP50 BeadChip (Illumina, SanDiego, CA). The final marker data included 48 073 SNPs for 5 214 bulls after removing SNP with minor allele frequency (MAF) less than 0.01 and locus average GenCall score less than 0.60.

De-regressed proofs (DRP) were used as response variables for genomic prediction in all approaches. Based on EBV data of 14 588 progeny-tested bulls and pedigree data of 42 144 animals, the de-regression was carried out by applying the iterative procedure described in [23,24] using the MiX99 package [25] and with the heritabilities shown in Table 1, which were those used in Nordic cattle routine genetic evaluation. A detailed description of the Nordic cattle genetic evaluation and standardized procedures of EBV is given in http://www.nordicebv.info/Routine+evaluation/.

### Statistical models

Three GBLUP and two single-step blending methods were used. All analyses were performed with the DMU package [26,27], for estimating both the variance components and breeding values.

#### *Simple GBLUP*

The basic GBLUP method [28,29] used to predict direct genomic breeding values (DGV) was:

(1) y=1μ+Zg+e

where **y** is the data vector of DRP of genotyped bulls, μ is the overall mean, **1** is a vector of ones, **Z** is a design matrix that allocates records to breeding values, **g** is a vector of DGV to be estimated, and **e** is a vector of residuals. It was assumed that $g∼N(0,G\sigma_{g}^{2})$ where $\sigma_{g}^{2}$ is the additive genetic variance, and **G** is the marker-based genomic relationship matrix [28,29]. Allele frequencies used to construct **G** were estimated from the observed genotype data. Random residuals were assumed such that $e∼N(0,D{\text{σ}}_{e}^{2})$ where ${\text{σ}}_{e}^{2}$ is the residual variance and **D** is a diagonal matrix with elements $d_{ii}=1/w_{i}$. The weights w_i_ account for heterogeneous residual variances due to differences in reliabilities of DRP. They were defined as $w_{i}{\text{=r}}_{i}^{2}{\text{/(1-r}}_{i}^{2})$, where $r_{i}^{2}$ is the reliability of DRP. The reliability was calculated as $r_{i}^{2}\text{=EDC/(EDC+k})$, where EDC is effective daughter contribution, and ${\text{k=(4-h}}^{2}{)\text{/h}}^{2}$. To avoid possible problems caused by extreme weight values, reliabilities larger than 0.98 were set to 0.98.

#### *GBLUP with a polygenic effect*

(2) y=1μ+Zu+Zg+e

where **u** is the vector of residual polygenic effects that are not captured by the SNP.

Here, we used an equivalent approach. Let $g_{\text{ω}}=u+g$, $\text{Var}(g_{\text{ω}})=A\sigma_{u}^{2}+G\sigma_{g}^{2}$, where **A** is the pedigree-based relationship matrix. Define $\sigma_{g_{\omega }}^{2}=\sigma_{u}^{2}+\sigma_{g}^{2}$ and $w=\sigma_{u}^{2}/(\sigma_{u}^{2}+\sigma_{g}^{2})$, then $w=\sigma_{u}^{2}/(\sigma_{u}^{2}+\sigma_{g}^{2})=\omega \sigma_{g_{\omega }}^{2}$ and $\sigma_{g}^{2}=(1-\omega )\sigma_{g_{\omega }}^{2}$, such that $\text{Var}(g_{\text{ω}})=[\text{ω}A+(1-\text{ω})G]\sigma_{g_{\omega }}^{2}$ where ω is the ratio of residual polygenic to total additive genetic variance. Thus, the above model is equivalent to

(3) $$y=1\text{μ}+Zg_{\text{ω}}+e.$$

It was assumed that $g_{\text{ω}}\sim N(0,\;G_{\text{ω}}\sigma_{g_{\omega }}^{2})$, where **G**_ω_ is a combined relationship matrix, $G_{\text{ω}}=\text{ω}A+(1-\text{ω})G$. The estimates of **g**_ω_ were defined as DGV_ω_ to distinguish from the simple GBLUP and the single-step blending methods.

#### *Adjusted GBLUP with a polygenic effect*

The model was the same as the above GBLUP method with a polygenic effect but **G** was adjusted to be on the same scale as **A**. Then, the combined relationship matrix was $G_{\omega }^{*}=\text{ω}A+(1-\text{ω})G^{*}$, where G* is the adjusted genomic relationship matrix**.** The adjustment of **G** is described below.

#### *Original single-step blending*

The original single-step blending method [15,17,18] uses information from genotyped and non-genotyped individuals simultaneously by combining the genomic relationship matrix **G** with the pedigree-based numerator relationship matrix **A**, using the following model:

(4) y=1μ+Za+e

where **y** is the vector of DRP for both genotyped and non-genotyped bulls, **1** is a vector of ones, **Z** is a design matrix, and **a** is the vector of additive genetic effects, which are the sum of the genomic and the residual polygenic effects. It was assumed that $a\sim N(0,H\sigma_{a}^{2})$, where matrix **H** is the modified genetic relationship matrix that combines pedigree-based relationship information [13,15]:

(5) $$H=[\begin{array}{l} G_{\omega }\\ A_{21}A_{11}^{-1}G_{\omega } \end{array}\;\begin{array}{c} G_{\omega }A_{11}^{-1}A_{12}\\ A_{21}A_{11}^{-1}G_{\omega }A_{11}^{-1}A_{12}+A_{22}-A_{21}A_{11}^{-1}A_{12} \end{array}]$$

where **A**_11_ is the sub-matrix of the pedigree-based relationship matrix (**A**) for genotyped animals, **A**_22_ is the sub-matrix of **A** for non-genotyped animals, **A**_12_ (or **A**_21_) is the sub-matrix of **A** for relationships between genotyped and non-genotyped animals, and $G_{\omega }=(1-\text{ω})G+\omega A_{11}$, where ω is a weight (within the range from 0.05 to 0.40 in this study). The **G** matrix used in the single-step blending was the same as in the GBLUP method. The inverse of **H**[15,17] is

(6) $$H^{-1}\text{=}\left[\begin{array}{cc} \begin{array}{c} G_{\text{ω}}^{-1}{-A}_{11}^{-1}\\ 0 \end{array} & \begin{array}{c} 0\\ 0 \end{array} \end{array}\right]{\text{+A}}^{-1}$$

#### *Adjusted single-step blending*

In the adjusted single-step blending method, the **G** matrix was adjusted for the difference between the original genomic relationship matrix and pedigree relationship matrix (**A**_11_), as proposed by previous studies [19,20]. The **G** matrix was adjusted using two parameters α and β [21], i.e.,

(7) G*=Gβ+α,

which were derived from the following equations:

(8) $$\text{Avg.diag(}G\text{)β+α=Avg}\text{.diag(}A_{11})$$

(9) $$\text{Avg.offdiag(}G\text{)β+α=Avg}\text{.offdiag(}A_{11})$$

Matrix G^*^ was then used to replace **G** to construct the combined relationship matrix in the single-step blending method.

The weights ω ranging from 0.05 to 0.40 were used to construct **G**_*ω*_ and $G_{\omega }^{*}$ for the single-step blending methods and for the GBLUP methods with a polygenic effect.

### Validation

The reliabilities of genomic predictions were measured as squared correlations between the predicted breeding values and DRP for bulls in the validation data, divided by the average reliability of the DRP in validation data. A Hotelling-Williams t-test was used to test the difference between the validation correlations obtained from these five prediction methods [30,31]. Bias of genomic predictions was measured as the regression of DRP on the genomic predictions [32].

## Results

Genomic predictions using the GBLUP method were improved when a polygenic effect was included (Tables 2 and 3). With a relative weight of 0.2 on the residual polygenic variance, the average reliability of genomic predictions for the 16 traits was 0.363, which was 0.3% points higher than the average reliability from the simple GBLUP. Moreover, the GBLUP method with a polygenic effect reduced bias of genomic predictions. Averaged over the 16 traits, the absolute deviation of the regression coefficient (DRP on genomic prediction) from 1 was 0.093 when using the GBLUP methods with a polygenic effect and 0.107 when using the simple GBLUP method. The GBLUP methods with a polygenic effect slightly reduced also bias in mean, as the intercept in the regression analysis was closer to 0, compared with the simple GBLUP. For the two GBLUP methods with a polygenic effect, adjustment of the genomic relationship matrix had no effect on predictive ability and bias.

**Table 2:** Reliabilities of genomic predictions using different methods

**Trait**	**GBLUP**	**GBLUP**_**AG**_	**GBLUP**_AG*_
Milk	0.431	0.428	0.428
Fat	0.455	0.457	0.457
Protein	0.429	0.435	0.435
Growth	0.468	0.481	0.481
Fertility	0.411	0.419	0.419
Birth index	0.258	0.263	0.263
Calving index	0.301	0.303	0.303
Mastitis	0.362	0.359	0.359
Health	0.435	0.435	0.435
Body conf.	0.313	0.316	0.316
Feet & Leg	0.311	0.307	0.306
Udder conf.	0.366	0.357	0.357
Milkingspeed	0.292	0.295	0.295
Temperament	0.184	0.183	0.183
Longevity	0.320	0.334	0.334
Yield	0.431	0.437	0.438
Mean	0.360	0.363	0.363

GBLUP without a polygenic effect (GBLUP), GBLUP with a polygenic effect and a weight of 0.2 (GBLUP_AG_), and adjusted GBLUP (i.e., using adjusted **G** matrix) with a polygenic effect and a weight of 0.2 (GBLUP_AG*_).

**Table 3:** Intercept (INT) and regression coefficient (REG) of DRP on genomic predictions from different methods

**Trait**	**GBLUP**		**GBLUP**_**AG**_		**GBLUP**_AG*_	
	INT	REG	INT	REG	INT	REG
Milk	2.028	0.920	1.455	0.960	1.445	0.961
Fat	2.837	0.877	2.385	0.912	2.377	0.913
Protein	3.906	0.847	3.182	0.883	3.169	0.884
Growth	−0.240	1.045	−0.246	1.083	−0.246	1.084
Fertility	1.439	0.980	1.583	1.032	1.586	1.034
Birth index	0.846	0.865	0.707	0.926	0.705	0.927
Calving index	1.002	1.016	0.822	1.060	0.819	1.061
Mastitis	0.365	0.937	0.283	0.947	0.281	0.947
Health	0.585	1.156	0.579	1.175	0.579	1.176
Body conf.	1.172	0.864	0.965	0.895	0.961	0.896
Feet & Leg	1.389	1.009	1.284	1.055	1.283	1.056
Udder conf.	2.973	0.899	2.705	0.926	2.701	0.926
Milkingspeed	1.751	0.836	1.575	0.886	1.572	0.887
Temperament	2.665	0.727	2.579	0.751	2.578	0.752
Longevity	2.537	0.905	2.171	0.939	2.164	0.940
Yield	3.975	0.853	3.286	0.887	3.273	0.887
Mean Dev.^1^	1.857	0.107	1.613	0.093	1.609	0.093

GBLUP without a polygenic effect (GBLUP), GBLUP with a polygenic effect with a weight of 0.2 (GBLUP_AG_) and adjusted GBLUP with a polygenic effect with a weight of 0.2 (GBLUP_AG*_); ^1^Mean of absolute deviation from 1 for regression coefficient and from 0 for intercept.

Table 4 reports validation reliabilities of GEBV from the two single-step blending methods and DGV_ω_ from the GBLUP method with a polygenic effect (the adjusted GBLUP method is shown as an example) for the 16 traits, with a relative weight ω = 0.20. The adjusted single-step blending led to the highest reliability of genomic predictions, followed by the original single-step blending, and the GBLUP method resulted in the lowest reliability. Reliabilities ranged from 0.206 to 0.503 (average 0.379) for the original single-step blending, from 0.206 to 0.503 (average 0.382) for the adjusted single-step blending, and from 0.183 to 0.481 (average 0.363) for the GBLUP method. In general, single-step blending was better than the GBLUP method and adjusted single-step blending was better than the original single-step blending, especially for production traits. On average, reliabilities of genomic breeding values predicted using the original single-step blending were 1.6 % higher than reliabilities from the adjusted GBLUP method, but 0.3% lower than reliabilities from the adjusted single-step blending.

**Table 4:** Reliabilities of genomic predictions using different methods

**Traits**	**GBLUP**_AG*_	**Single**_**ori**_	**Single**_**adj**_	**Single**_**ori**_**-GBLUP**_AG*_	**Single**_**adj**_**-GBLUP**_AG*_	**Single**_**adj**_**-Single**_**ori**_
Milk	0.428	0.450	0.456	0.022^*^	0.028^**^	0.006^*^
Fat	0.457	0.458	0.466	0.001	0.009^*^	0.008^**^
Protein	0.435	0.437	0.446	0.002	0.011	0.009^**^
Growth	0.481	0.503	0.503	0.022^**^	0.022^**^	0.000
Fertility	0.419	0.425	0.431	0.006	0.012	0.005
Birth index	0.263	0.274	0.274	0.011	0.011	−0.001^*^
Calving index	0.303	0.328	0.329	0.025^**^	0.026^**^	0.002
Mastitis	0.359	0.383	0.384	0.024^**^	0.025^**^	0.000
Health	0.435	0.467	0.469	0.032	0.034^*^	0.003
Body conf.	0.316	0.317	0.317	0.001	0.001	0.000
Feet & Leg	0.306	0.296	0.296	−0.01	−0.01	0.000
Udder conf.	0.357	0.358	0.358	0.001	0.001	−0.001
Milkingspeed	0.295	0.312	0.312	0.017^*^	0.017^*^	0.000
Temperament	0.183	0.206	0.206	0.023^*^	0.023^*^	0.000
Longevity	0.334	0.415	0.415	0.081^**^	0.081^**^	0.000
Yield	0.438	0.436	0.446	−0.002	0.008	0.010^**^
Mean	0.363	0.379	0.382	0.016	0.019	0.003

Adjusted GBLUP with a polygenic effect with a weight of 0.2 (GBLUP_AG*_), original single-step blending (Single_ori_) and adjusted single-step blending (Single_adj_) with a weight of 0.2; ^*^significant difference at p < 0.05; ^**^significant difference at p < 0.01.

The regression coefficients (Table 5) ranged from 0.757 to 1.138 (average absolute deviation from 1 equal to 0.084) for the original single-step blending, from 0.760 to 1.148 (average absolute deviation 0.080) for the adjusted single-step blending, and from 0.752 to 1.176 (average absolute deviation 0.093) for the adjusted GBLUP method. Predictions from the single-step blending methods appeared to have less bias than predictions from GBLUP, and predictions from the adjusted single-step blending has slightly less bias than predictions from the original single-step blending method. In addition, the two single-step blending methods led to smaller absolute deviation of the intercept from 0 than the adjusted GBLUP method, indicating less bias in mean.

**Table 5:** Intercept (INT) and regression coefficient (REG) of DRP on genomic predictions using different methods

**Trait**			**GBLUP**_AG*_		**Single**_**ori**_		**Single**_**adj**_	
	INT	REG	INT	REG	INT	REG	
Milk	1.445	0.961	1.225	0.963	0.843	0.975
Fat	2.377	0.913	2.136	0.910	1.752	0.932
Protein	3.169	0.884	2.967	0.877	2.441	0.898
Growth	−0.246	1.084	−0.133	1.093	−0.103	1.095
Fertility	1.586	1.034	1.633	1.023	1.917	1.044
Birth index	0.705	0.927	0.608	1.054	0.583	1.057
Calving index	0.819	1.061	0.439	1.009	0.520	1.019
Mastitis	0.281	0.947	0.206	0.954	0.246	0.958
Health	0.579	1.176	0.677	1.138	0.793	1.148
Body conf.	0.961	0.896	0.652	0.913	0.605	0.918
Feet & Leg	1.283	1.056	1.058	1.028	1.051	1.030
Udder conf.	2.701	0.926	2.144	0.934	2.114	0.935
Milkingspeed	1.572	0.887	1.371	0.858	1.355	0.861
Temperament	2.578	0.752	1.816	0.757	1.795	0.760
Longevity	2.164	0.940	1.531	0.963	1.384	0.969
Yield	3.273	0.887	3.079	0.878	2.524	0.902
Mean Dev.^1^	1.609	0.093	1.355	0.084	1.252	0.080

Adjusted GBLUP with a polygenic effect with a weight of 0.2 (GBLUP_AG*_), original single-step blending (Single_ori_) and adjusted single-step blending (Single_adj_) with weight a weight of 0.2; ^1^Mean of absolute deviation from 1 for regression coefficient and from 0 for intercept.

Table 6 presents differences between groups of the top 300 bulls based on predictions from the different methods. For all 16 traits, more than 9% of the top 300 bulls based on the adjusted GBLUP method differed from the top 300 bulls based on the two single-step blending methods. Differences between the two single-step blending methods were small, except for production traits, which was in agreement with the small differences in reliabilities of GEBV from the two single-step blending methods.

**Table 6:** Differences between groups of the top 300 bulls based on genomic prediction using different methods

**Trait**	**GBLUP**_AG*_Vs. Single_**adj**_	**GBLUP**_AG*_Vs. Single_**ori**_	**Single**_**ori**_**Vs. Single**_**adj**_
Milk	39	38	18
Fat	33	33	11
Protein	36	38	17
Growth	42	44	3
Fertility	29	33	8
Birth index	32	32	2
Calving index	38	39	4
Mastitis	32	33	1
Health	33	35	6
Body conf.	32	31	3
Feet & Leg	36	37	2
Udder conf.	38	40	3
Milkingspeed	35	35	1
Temperament	48	46	2
Longevity	41	44	8
Yield	27	31	16

Adjusted GBLUP with a polygenic effect with a weight of 0.2 (GBLUP_AG*_), original single-step blending (Single_ori_), and adjusted single-step blending (Single_adj_) with a weight of 0.20, measured as the number of bulls that are not among the top 300 based on the second method.

**Figure 1:**
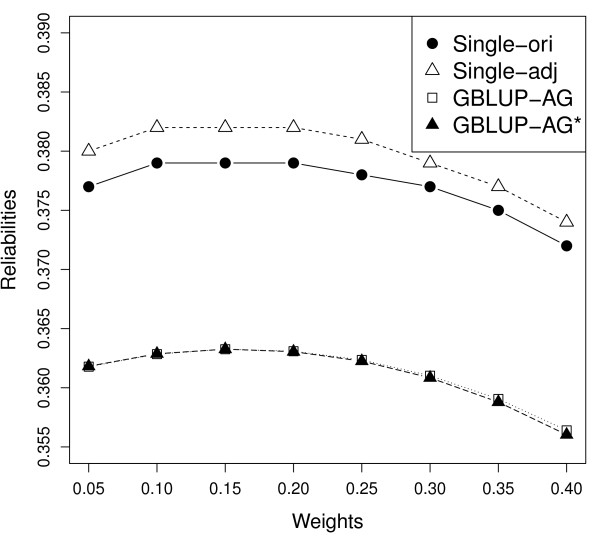
**The impact of different weights on reliability of genomic predictions using different methods.** GBLUP with a polygenic effect (GBLUP-AG), adjusted GBLUP with a polygenic effect (GBLUP-AG^*^), original single-step blending (Single-ori), and adjusted single-step blending (Single-adj).

**Figure 2:**
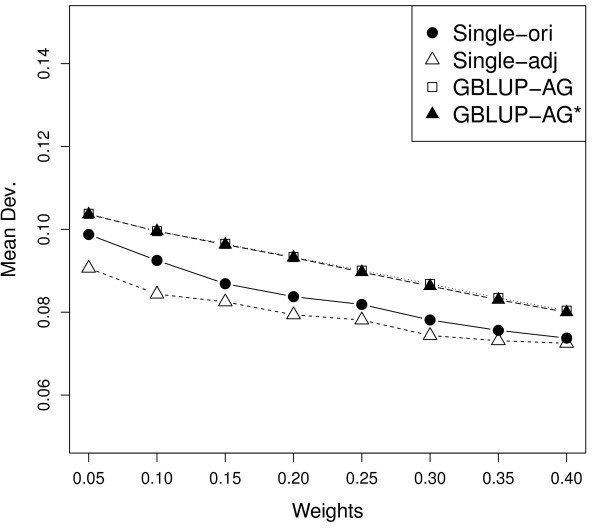
**The impact of different weights on the mean absolute deviation from 1 of the regression coefficient of DPR on prediction using different methods.** GBLUP with a polygenic effect (GBLUP-AG), adjusted GBLUP with a polygenic effect (GBLUP-AG^*^), original single-step blending (Single-ori), and adjusted single-step blending (Single-adj).

In order to test the effect of different weighting factors ω in forming **G**_ω_ and **H**, eight values of ω between 0.05 and 0.40 were used for the two single-step blending methods and the two GBLUP methods with a polygenic effect. On average, reliabilities varied from 0.356 to 0.363 over the eight scenarios for the two GBLUP methods, from 0.372 to 0.379 for the original single-step blending, and from 0.374 to 0.382 for the adjusted single-step blending (Figure 1). The highest mean reliability was obtained when using a weight of 0.15 or 0.20 for the four methods. The mean absolute deviation of the regression coefficient from 1 varied from 0.080 to 0.104 for the two GBLUP methods, from 0.074 to 0.098 for original single-step blending and from 0.072 to 0.091 for adjusted single-step blending (Figure 2). Mean of absolute deviations tended to decrease with increasing weights.

## Discussion

This study applied three GBLUP and two single-step blending methods for genomic prediction in Nordic Holsteins. Predictive abilities of the five methods were compared in terms of reliability and bias. Results indicated that both the original single-step blending and the adjusted single-step blending were more accurate than the three GBLUP methods because the two single-step blending approaches used much more information to predict breeding values. Similar results were reported by Su et al. [18] for the Nordic Red population. In the current study, the size of the training dataset for the single-step blending methods was almost three times as large as that for the three GBLUP methods (Table 1) since DRP of the non-genotyped animals also provided information through a combined relationship matrix. Including pedigree information may also improve genomic predictions because the SNP may not account for all additive genetic variance. As shown in this study, including a residual polygenic effect in the GBLUP methods led to slightly higher reliability of genomic predictions.

A regression coefficient of DRP on genomic predictions less than 1 indicates overestimation of the variance of genomic predictions (inflation), while a coefficient larger than 1 indicates underestimation (deflation). The two single-step blending methods led to less bias than the three GBLUP methods, and the two GBLUP methods with a polygenic effect resulted in less bias than the simple GBLUP method without a polygenic effect. The problem of inflation of genomic predictions is critical in practice [33-35] as it can give an unfair advantage to juvenile over older progeny test bulls [17]. Aguilar et al. [17] showed that this bias was reduced by weighting the **G** and **A** matrices, and Liu et al. [36] found that including a polygenic effect in a GBLUP model (random regressions on SNP genotypes) led to less bias in genomic predictions. The present study showed that the weighting factor had an effect on the bias of genomic predictions for all traits in the single-step blending approaches and the GBLUP methods with a polygenic effect. A weight of 0.40 resulted in the smallest minimum absolute deviation from 1 for the regression of GEBV or DGV_ω_ on DRP, averaged over the 16 traits, but a loss of reliability around 0.8%, compared to a weight of 0.20, which led to highest average reliability and an acceptable average absolute deviation of regression coefficient from 1 (Figure 1,2).

The adjusted single-step blending method resulted in less bias than the original single-step blending for all settings of the weight factor. In a simulation study, Vitezica et al. [19] also found that the single-step method was less biased and more accurate when the genomic relationship matrix was adjusted by a constant. Using chicken data, Chen et al. [20] showed that unbiased evaluations can be obtained by adding a constant to the **G** matrix that is based on current allele frequencies and suggested that the optimal **G** has average of diagonal and off-diagonal elements close to those of **A**_11_. Forni et al. [22] also showed that re-scaling the **G** matrix is a reasonable solution to avoid inflation in pig data. However, in the present study, the adjusted **G** matrix did not improve genomic predictions in the GBLUP methods with a polygenic effect. This suggests that, based on the present data, adjustment of **G** has little effect on genomic prediction when only genotyped animals are used, but may be important in other data where there is a large difference in scale between **G** and **A**.

The results from the present study indicate that increasing the weighting factor (0.40) reduces bias and that weighting factors around 0.15 to 0.20 give the highest reliability but the optimal weighting factors differed between traits. Similarly, Liu et al. [36] observed that the optimal residual polygenic variance in a GBLUP model (random regressions on SNP genotypes) with a polygenic effect appears to differ among traits. Therefore, trait-specific weighting factors should be used in the single-step blending methods and the GBLUP methods with a polygenic effect. In the near future, both bulls and heifers may be pre-selected based on genomic EBV. This will lead to biased predictions of breeding values in both conventional and genomic evaluation procedures. In such situations, appropriate methods to correct the bias of predictions are required [37].

Christensen et al. [21] compared the adjusted and original single-step blending methods on pig data. In their study, the improvement of prediction reliabilities by adjustment of **G** matrix is much larger, compared with the results from the current study. This may be because there was more inbreeding in the pig data, which resulted in average values of the diagonal and off-diagonal elements of **A**_11_ equal to 1.145 and 0.298, and estimates of β and α equal to 0.895 and 0.298, respectively. In the present study, the averages of the diagonal and off-diagonal elements of **A**_11_were 1.060 and 0.085, and estimates of β and α were 0.976 and 0.085, i.e. closer to one and zero, respectively. This means that the original **G** matrix was less adjusted in this study compared to the study on pig data by Christensen et al. [21].

## Conclusions

The single-step blending methods can increase reliability and reduce bias of genomic predictions. The adjusted single-step blending method performed slightly better than the original single-step blending method, both with respect to reliability and bias of genomic predictions. The weighting factor used in these single-step blending methods had a small effect on reliability of genomic prediction but an important effect on bias.

## **Competing interests**

The authors declare that they have no competing interests.

## **Authors’ contributions**

HG performed statistical analysis and wrote the manuscript. OFC derived the single-step methods and improved the manuscript. PM provided the software, helped to the analysis and added valuable comments. USN prepared the data. GS and MSL conceived the study, made substantial contribution for the results interpretation and revised the manuscript. MSL, GS and YZ coordinated the project. All authors read and approved the manuscript.
